# Sutureless off-pump repair of post-infarction left ventricular free wall rupture

**DOI:** 10.1186/1749-8090-1-11

**Published:** 2006-05-18

**Authors:** Hunaid A Vohra, Samena Chaudhry, Christopher MR Satur, Mary Heber, Rob Butler, Paul D Ridley

**Affiliations:** 1 Department of Cardiothoracic Surgery, University Hospitals North Staffordshire NHS Trust, Stoke-on-Trent, UK; 2 Department of Cardiology, Princess Royal Hospital, Telford, UK; 3 Department of Cardiology, University Hospitals North Staffordshire NHS Trust, Stoke-on-Trent, UK

## Abstract

Left ventricular free wall rupture after myocardial infarction has a high mortality. Suturing techniques of repair may be technically difficult and require cardiopulmonary bypass. We report a case of left ventricular rupture in a 47 year old man managed off pump employing a sutureless technique with Gelatine-Resorcin-Formalin glue and bovine pericardial patches.

## Introduction

Left ventricular free wall rupture post myocardial infarction has a high mortality and therefore, rarely presents to the cardiac surgeon. Conventional approaches to this condition include ventricular repair with teflon buttressed sutures using cardiopulmonary bypass (CPB). Coronary artery bypass grafting may be performed concomitantly. Some authors have suggested sutureless techniques with or without cardiopulmonary bypass for this condition. We report a case of off-pump repair with bovine pericardial patches using Gelatine-Resorcin-Formalin (GRF) glue.

## Case report

**Figure 1:**
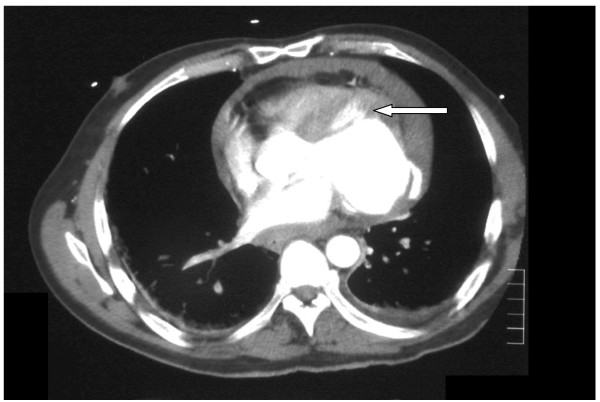
CT scan showing leakage of contrast from the left ventricle free wall rupture (arrow).

A 47 year old man presented to the Emergency department with central chest pain. Lateral myocardial infarction (MI) was confirmed by typical changes in leads I, AVL and V6 and raised troponin T levels. The patient was not thombolysed. He was haemodynamically stable initially and a coronary angiogram was contemplated. However, before this could be performed, he developed further chest pain and became haemodynamically unstable with tachycardia, hypotension and collapse (at 48 hours). An urgent trans- thoracic echocardiogram (TTE) showed a pericardial effusion. Left ventricular (LV) free wall rupture was suspected. Contrast-enhanced computed tomography (CT) of the chest was performed which confirmed this, as well as a large pericardial effusion (figure 1). He was transferred from the referring hospital to the nearest cardiothoracic centre (one hour journey) for surgical repair. On arrival, it was felt that he would not have survived a trip to the catheter lab. A peri-operative trans-oesophageal echocardiogram (TOE) demonstrated pericardial tamponade and left ventricular free wall rupture in the region supplied by the circumflex artery. Surgery was performed via a median sternotomy. A large amount of blood and clot was removed from the pericardium with immediate haemodynamic improvement. A large recent infarction was identified involving the obtuse margin of the heart. This part of the myocardium was typically oedematous and fragile. At the centre of the infarcted area blood spurted from a small hole in the left ventricular free wall. The pericardium was sutured bilaterally to the skin edges and the heart was gently lifted placing large swabs behind it for surgical exposure. Surgery was performed on the beating heart without the use of cardiopulmonary bypass. The LV wall defect was repaired with Gelatine-Resorcin-Formalin (GRF glue: Cardial, Saint-Etienne, France) applied to a 6 cm diameter bovine pericardial patch (Impra, Tempe, Arizona, USA). This sutureless technique was repeated four times using overlapping patches of bovine pericardium to ensure coverage of the surrounding infarcted area of the left ventricular free wall. After ensuring adequate hemostasis, the heart was replaced into the pericardial sac. An intra-aortic balloon pump (IABP, Datascope, Oakland, NJ, USA) was inserted electively after the procedure to reduce cardiac after-load. Post-operatively, the patient returned to the intensive therapy unit in a stable condition without inotropic support. The patient was successfully extubated twelve hours post-operatively and remained haemodynamically stable. Loss from the chest drains was modest (440 mls in 24 hours). The IABP was removed two days after surgery. The rest of the post-operative course was uneventful. There was no evidence of end-organ injury. A pre-discharge trans-thoracic echocardiogram did not show any leak of blood across the ventricular repair. There was minimal pericardial effusion and moderate left ventricular ejection fraction with no valvular abnormalities. The patient was discharged home on the fifth post-operative day. Subsequent investigation with coronary angiogram and magnetic resonance imaging scan have shown complete occlusion of the circumflex coronary artery. Irregularities were seen in the left anterior and right coronary arteries but there were no significant stenoses in these vessels. The obtuse margin of the heart was akinetic and the left ventricle was dilated with overall moderate function. At eighteen month follow up the patient is clinically well being active and experiencing only mild dyspnoea on exertion (NYHA II).

## Discussion

Myocardial infarction complicated by left ventricular free wall rupture and pericardial tamponade occurs in 2–4% of cases and is associated with a high mortality [1]. Autopsy studies reveal that left ventricular free wall rupture (LVFWR) accounts for 5–24% of deaths after myocardial infarction [1]. The condition occurs up to ten times more often than papillary muscle rupture or interventricular septal rupture. A high index of suspicion must be maintained to differentiate left ventricular free wall rupture from infarct extension, cardiogenic shock, pulmonary embolus and even Dressler's syndrome. Classically, left ventricular free wall rupture occurs in patients aged between 65 and 70 years. It should be strongly suspected with presenting features like continued chest pain, acute arterial hypotension and persistent ST elevation.

Traditional left ventricular free wall rupture repair techniques such as a series of interrupted pledgeted sutures, reinforced linear teflon strips and circular patch repair often involve tying sutures though friable necrotic muscle [2]. Heparinisation with CPB may lead to continuous oozing of blood though necrotic areas of myocardium adjacent to the primary site of infarction. Recent thrombolysis might also contribute to a coagulopathy in these patients. In the paper by Padro et al [3] in 13 patients with left ventricular free wall rupture after myocardial infarction, a teflon patch was applied over the ruptured area and glued to the heart surface with a cyanoacrylate glue without the use of cardiopulmonary bypass. Follow-up extending up to 5 years (mean = 26 months) showed a 100% survival. Lijoi et al [4] also reported a sutureless off-pump technique on two patients using a teflon patch fixed to the ventricular wall with a biocompatible synthetic glue, ethyl-2-cyanoacrylate monomer. The two patients survived the operation and were discharged from the hospital 12 and 14 days after surgery.

Mariani et al [5] described an off-pump sutureless technique with the use of a glutaraldehyde-treated bovine pericardial patch and a synthetic biocompatible glue (N-butyl-2-cyanoacrylate) in a 60-year-old male patient with echocardiographic evidence of cardiac tamponade and a free wall rupture site on the lateral wall. The patient was discharged from the hospital on the eleventh postoperative day. Mantovani et al [6] report seventeen patients who underwent underwent surgery for ventricular free wall rupture. Patch covering technique with glue was used in 13 patients (Gelatine-Resorcin-Formalin glue in 11 patients and fibrin glue in 2 patients), infarctectomy with patch reconstruction in three patients and direct suture without patch in one patient. Three patients died of cancer during the follow-up. The remaining 11 patients were in good condition after a mean follow-up of 45.8 months. Their first choice for repair was reported as being the covering technique with a large pericardial patch anchored with biological glue and epicardial sutures.

The traditional approach is to proceed with infarctectomy and replacement with a prosthetic patch under CPB [2]. Prior to the popularization of the patch glue repair, there had been little improvement in survival with infarctectomy and repair utilizing CPB. Although no direct comparative studies have been performed, results with the patch glue technique appear to be superior [2-6].

Basically, there are two types of glue: synthetic (most commonly cyanoacrylates) and biological (GRF, fibrin, cryoprecipitate, etc). Degradation of the cyanoacrylates can lead to tissue toxicity. The shorter-chain compounds (butyl) tend to have a higher degree of tissue toxicity than the longer-chain derivatives (octyl). These adhesives work by polmerising after which the adhesive may become brittle and is subject to fracturing. This is especially true in areas of high tension. These limitations of synthetic glues lead to the development of biological glues. The GRF glue should be used as a tissue reinforcer, the two-component fibrin sealer is preferable when haemostatic action must be accompanied with mechanical barrier and the cryoprecipitate glue can be used when haemostatic action is the only requirement.

It is not known whether the benefit of CABG is worth the inherent risk involved in delaying definitive surgical therapy by performing coronary angiogram. In a review of literature, only 9 of the 87 long term survivors of free wall rupture repair had CABG as part of their surgical intervention [7]. Nakamura et al [8] have shown that the incidence of cardiac rupture after myocardial infarction is marginally lower (1.7% in 533 patients versus 2.7% in 807 patients) in patients who undergo thrombolysis. They also showed that early reperfusion therapy significantly reduced the incidence of ventricular rupture on or after 4 days, but not within 24 hours.

On the basis of our case report and the current literature we can say that left ventricular free wall rupture after myocardial infarction can potentially be repaired without the use of extra-corporeal circulation using the sutureless technique with glue and pericardial patch. Gluing is an alternative to suturing in such a desperate situation.
